# Clinical Lecture

**Published:** 1886-04

**Authors:** Theophilus Parvin

**Affiliations:** Philadelphia


THE BUFFALO MEDICAL AND SURGICAL JOURNAL

VOL. XXVI. APRIL, 1886. No. 9.

### Original Communications.

### CLINICAL LECTURE.

By Prof. THEOPHILUS PARVIN, M. D., of Philadelphia.

Delivered at the Philadelphia Hospital, February 10th.

### ACCIDENTAL HEMORRHAGE.

I have the opportunity to-day, gentlemen, of bringing before you a very interesting and instructive case. This German woman, aged 21, a domestic by occupation, and a widow, has always enjoyed good health. She has no family history of any kind that is unfavorable. She has had no miscarriages, and has one child living, who is now three years old. Her last menstruation occurred June 20th last, since which time she has been pregnant. Last Saturday, while doing some work, she slipped and fell, striking the right iliac region against the edge of a stove. There was, as a consequence of this fall, great pain in the abdomen and very free hemorrhage, and the fœtal sounds could not be heard. She was at once put to bed, and appropriate treatment (which I shall refer to later) instituted. By the next day the hemorrhage had ceased, and the murmurs could be heard. I might say that a trace of albumen was found in the urine.

Now, this case is possessed of very great clinical interest. If you will make a calculation from the date that I have indicated, you will observe that the woman ought to be confined about the 25th of March. Here, then, we have accidental hemorrhage, occurring very copiously in the eighth month of gestation. It is not so uncommon to meet with hemorrhage during the latter months of pregnancy, which may be caused, as in this case, by a fall, or may be brought about by a simple jar or contusion of the uterus. For centuries we have had discussions going on in reference to this question of accidental hemorrhage, as to its causation, what should be called accidental hemorrhage, and especially in reference to the best treatment. It is a somewhat curious and somewhat inexplicable fact that accidental hemorrhage is frequently associated with albuminuria; in fact, it is quite as noticeable as is the association of albuminuria with post-partum hemorrhage. From this fact, we must draw the inference that when we find albumen in the urine of a pregnant woman, we must bear in mind, as one of the possibilities of this condition, the occurrence of accidental hemorrhage. The condition of the blood, in such cases, seems to exert a predisposing influence. Some few years ago, Dr. Goodell, of the University, collected and made tabular statistics of 106 cases of accidental hemorrhage, in which there were 107 children. From these tables we learn the startling facts, that of the women there was a mortality of over 50 per cent., and of the 107 children, only six were born alive. From this we can see that the occurrence of this hemorrhage is a matter of very grave import, though I think the actual danger, especially to the child, is somewhat exaggerated when it occurs, as it has in this case, during the eighth month. Now, when we have hemorrhage—and by this word I mean *hemorrhage*, and not simply a *bleeding* or an *oozing*—we may know that the placenta has become partially detached. In this fact we have the distinction between accidental and unavoidable hemorrhage; in accidental, the placenta is in its normal position, and some portion of its attachment becomes detached by violence, while in unavoidable hemorrhage, the placenta is attached somewhere to the lower segment of the uterus. Recently the effort has been made to limit the term unavoidable hemorrhage to that which occurs in labor. Now, I think the most general view is, that the shortening of the cervix begins during the last six weeks of pregnancy. According to the views of some, the uterus, in this period, grows away from the placenta, while others hold that the placenta grows away from the uterus. Now, I lean towards the view that the uterus grows away from the placenta, because we know that the placenta does not grow at all during the last few weeks of pregnancy. Now, this hemorrhage we can here trace to a direct and certain cause; we know it is not caused as in placenta prævia.

Well, now, this woman's pale face indicates that the hemorrhage has brought about a marked condition of anæmia. Her pulse is intermittent, and last night it was 110°. This frequency of the pulse is an expression of impoverished blood. If, at your boarding-house, you were fed on dish-water, you would be obliged to pass up your plate very often for this attenuated nutriment, that you might get enough to maintain life. So is it with this attenuated blood. The heart must act more rapidly to send a greater supply to the tissues, in order that what the blood lacks in quality may be made up in quantity. Now, let us endeavor to ascertain to what extent the placenta has been detached, for this will have an important bearing on our prognosis. If, upon auscultation, we find that the fœtal heart sounds do not vary very much in character and frequency from the normal, then we may rest assured that a considerable portion of the placenta is still attached, enough to carry on the fœtal circulation, to oxygenate the blood of the fœtus, and the chances are, therefore, that labor will not be interfered with. In some cases the hemorrhage may be concealed. The placenta may remain attached all around its circumference, while in its center it is separated; thus the blood will collect between the placenta and the uterus, and offer no outward manifestation of what is going on within. In such cases we will have the symptoms of shock from loss of blood, superadded to which will be the presence of a sensitive tumor in the abdomen. It is easy enough to make the diagnosis in a case like this, where we have external hemorrhage; but it is quite different when it is concealed. In the latter contingency, if we have the history of an injury, it will aid us, particularly if with shock; we have, as I have said, a tumor in the abdomen, a bulging up, as it were, one convexity on top of another. Such symptoms might also be present in rupture of the uterus, save the tumor, which we would not have.

Well, now, how shall we treat accidental hemorrhage? Some authorities will advise you to at once rupture the membranes and complete delivery. The child is viable, they will say, and this is your course. But I hold that such a course is justifiable only when all other means have failed. It is true that the child may be said to be viable at six and a half or seven months, that it is then capable of maintaining an independent existence; but I say that it is only relatively, and not positively, viable at this early date. That is to say, a child born at six and a half months will not have so good a chance of survival as one born at seven months; the seven months' fœtus will not be as vigorous as that at the eighth, and so on, so that, unless the life of the mother is at stake, we are not justified in taking this risk with the life of the child. Now, in this case, the external os is so patulous that the finger can be passed through it up to the internal os, but the membranes cannot be touched; by a little pressure, however, I think we could reach them. This condition of the os is not uncommon, even in primipara. The cervical canal here is about one inch long. I am in the habit of saying that as long as there is a cervical canal, the woman is pregnant; when it has become obliterated, she is in labor. I know of no more positive way of distinguishing between pregnancy and labor. This woman has a cervix, therefore she is not in labor; so I do not think it would be wise, in this case, to rupture the membranes and complete delivery. Well, then, what was done for her? She was placed at absolute rest, on her back, in bed—rest by recumbency and rest by the use of opium—and future developments were awaited. Now, it is an interesting question to know what happens to this portion of the placenta that is detached. After labor, we will find it comparatively smooth on the uterine surface and healed over, while the remainder will be rough and irregular, as we ordinarily see it. Nature will have occluded the orifices of the vessels, and will have smoothed over the surface. The woman will, in probability, go on safely to full term. Let this case be firmly impressed on your mind as an argument in favor of the temporizing treatment of accidental hemorrhage. This patient lost fully a quart of blood, yet she will go on to full term. Playfair stands prominently among those who advocate the termination of pregnancy in such conditions, but I say let the woman go on if it is at all within the bounds of possibility. Even in the most severe cases of hemorrhage, I would not advise you to interrupt pregnancy at once. If the hemorrhage persists in spite of rest and opium, then, as I have said, it may become necessary to do so; but when we learn that 50 per cent. of the mothers and 101 per cent. of the children die, we surely cannot find much encouragement for that practice.

### LOSS OF FLESH IN PREGNANCY—TUBERCULOSIS VS. PREGNANCY.

When a woman loses flesh during pregnancy, gentlemen, you have very strong reason to suspect that there is something wrong. The rule is, that during the last three months of pregnancy, a woman should gain one-thirteenth of her weight. When she does not do so, when she stands still, or still more so, when she loses weight, depend upon it, there is a screw loose somewhere. Well, now, this woman is twenty-eight years old, and this is her second pregnancy. Upon inquiry into her family history, we learn that her father died of consumption, while her mother and four sisters are living and well. She herself was perfectly well until three years ago, when she tells us that she had some heart trouble. Now, she has been in this hospital for ten weeks, and for eight weeks she has had a persistent and annoying cough. One week ago, I ordered her a prescription, containing morphia and hydrocyanic acid, since which time the cough has been relieved. This cough had caused great soreness in the abdomen, which is also better since the relief of the cough. The apex beat of the heart is felt in the nipple line, while in the mitral area we have a pre-cystolic murmur and a thrill. There is dullness on percussion over the upper portion of the left lung anteriorly, and bronchial rales heard all over the chest. Now, I want you to remember particularly the soreness of the abdomen, which might prove an important complicating factor in labor, for nature heeding the outcries of additional pain caused by this sore abdomen might respond by lessening the contractions of the uterus to relieve it, and thus labor be interfered with. Hence, it is of great importance that the cause of the soreness should be removed. Do not let a woman cough during the latter part of pregnancy, if you can possibly avoid it. So, also, this concussion may be the means of inducing premature labor. Well, in this case we have the physical signs of consolidation of the left lung. The pulse is 96, but, rather strange to say, there has been no evening rise of temperature. But this might be in part explained by the fact that there is very little expectoration. Respiration is more rapid than usual.

This case opens up the interesting question of the relation of tuberculosis to pregnancy. The late Prof. George B. Wood, who was one of the greatest teachers we have ever had, was wont to teach that it was a benefit to those who were predisposed to tuberculosis to get married, and he illustrated his advice by citing many cases where such benefit had accrued. In such cases, while the woman kept on bearing children, one pregnancy after another, the progress of the disease seemed to be held in check, but when they ceased having children the disease advanced with rapid progress. Some authorities who share this view, explain its occurrence by reasoning that pregnancy causes a diversion of the blood from the chest to the uterus, and so retards the process of disease in the chest. On the other hand, there are those who hold that pregnancy hastens the development of tuberculosis, and finally there are those who have a mixed opinion ; that is, that while it hastens it in some, it retards it in others. For myself, I think it may be stated that the disorder of the system, the general storm. the more rapid circulation of pregnancy, have a tendency to rouse the dormant disease into activity. So, therefore, I could not conscientiously recommend matrimony as a prophylactic. It is only, I think, the strong and well who are justified in taking upon themselves the grave responsibility of becoming fathers and mothers, and especially should such be avoided by those who already have or are inclined towards pulmonary disease. It would be better for society at large if this rule was observed. Maternity is a trial for the strongest constitution ; pregnancy is a nine months' voyage. It may be compared to a ship sailing off to a distant point ; she will encounter adverse winds, storms and tempestuous weather ; if she be strong and well built, she will weather the storms and ultimately reach her destined port in safety ; but if she be weak and strained, if she be infirm and leaky, she will find a watery grave in the bottom of the sea, cast down by the fury of the elements which she was not strong enough to defy. So is it with the pregnant woman ; her safety depends upon the integrity of all her parts, and if there be one weak organ, the strain of pregnancy will magnify and intensify this weakness. So, therefore, I must needs look upon it as a misfortune when a woman with pulmonary tuberculosis, or one predisposed thereto, becomes pregnant. We cannot evolve the clean from that which is unclean. Can we hope to have healthy offspring from a diseased parentage? It is not probable. In a large percentage of such cases, there will be a premature labor or a miscarriage, which seems to be Nature's method to prevent the perpetuation of a diseased offspring. In some cases that I can recall, the women did fairly well. They had apparently large, healthy children, but after labor they broke down completely, and hastened to a fatal termination. The moral, to my mind, is that such persons should not be reproducers. If you have a case of pregnancy in a woman who is predisposed to tuberculosis, you should watch her very carefully and be prepared to treat the very first symptoms. In this case the cough is better, as I have told you. I would also like to give this woman cod liver oil, if her stomach will tolerate it, and I would like her to drink two or three pints of milk daily. It is a fact that pulmonary tuberculosis is very common among females, and I think I might say that one-third of all the cases occurring in adult married life have their inception in miscarriages or after labor.

### PUERPERAL SEPTICÆMIA.

I had hoped to have shown you the case about which I am going to make some remarks, but she is not well enough for me to venture bringing her from the ward. I wish to call your attention to a very significant fact in reference to the influence that has been exerted over puerperal septicæmia in this hospital, by the factors of better wards for lying-in women and better nursing, which mean, in one word, greater *cleanliness*. By greater cleanliness marvellous results have been achieved. I happen to have been on duty in this hospital, for several years past, at that season of the year when puerperal septicæmia is most rife. Three years ago we had a great number of cases of a most virulent type, this year we have only three, last year we had ten or twelve; this year we have had no deaths, last year we had two. Therefore, I believe, that if in time the disease that has been the bane of this hospital is not entirely stamped out, we will at least have but an occasional case. The patient, to whom I have referred, was confined about eight weeks ago, she was a primipara, and there was retention of the placenta, from what is called hour-glass contraction. Now, according to some authorities, this hour-glass contraction of the uterus is due to a contraction of the circular fibres, about the middle of the organ, while others maintain that it is simply a normal contraction of the internal os, above which the placenta is imprisoned. Well, what would you do if there were no symptoms, yet the placenta had not come away? Why, take off your hat and wait. I do not know how better to advise you. This is what Henry Ward Beecher says he always feels like doing to a railroad train. And it is what I always feel like doing to a uterus, when the placenta fails to come away promptly. If there were hemorrhage, of course, I would interfere; but I say emphatically, gentlemen, that I always tremble when I contemplate passing my finger into a uterus that has been emptied of its fœtus. Before the fœtus has been expelled, it is a comparatively trivial matter to pass the finger, or even the whole hand, even up to the fundus, for the organ is sheathed by its membranes, and you do not come directly into contact with it. But after the fœtus has passed away, when there are gaping sinuses, tears, more or less, in the cervix, abrasions of vagina and vulva, all like so many hungry wolfs, eager to grasp any germs of disease that may be about your finger,—I say, I tremble when I realize what terrible consequences may result to the woman from the simple introduction of the finger. The placenta is normally delivered by a retraction of the womb, by which the surface of attachment is gradually lessened, and it finally becomes totally detached. Baudelocque has been accustomed to describe it as a pushing off, but it is not, it is a gradual and universal separation. So you can realize that the harder, the firmer, the placenta is, the more readily it is separated. If it be limp it will follow, so to speak, the retractions of the uterus, will maintain its adherence, and will not be detached so readily. Hence I always apply two ligatures to the cord, so that the placenta may not be made limp and flaccid by the drainage of blood that will occur if only one ligature be used. Lincoln was wont to say that it was easier to pay small debts than large ones, and those who allow the cord to drain, argue that the placenta is thus more readily delivered, because it is thus reduced in size. But I think this argument is fallacious, because, by folding on itself, the size of the placenta is much diminished. Well, then, in this case the placenta was not delivered. When you resort to artificial detachment, it is next to impossible to remove every particle; some will remain fixed, and it must be a terrible necessity that will ever drive a doctor to try to remove all. Two years ago, in this house, an adherent placenta was detached by the hands. In a short time the woman was seized with septicæmia, and so were her neighbors on either side. So, I say, look with fear on this procedure.

On the third day after labor, this patient had a chill, and her temperature has been persistently high, reaching nearly to 106°. Three days after the manual extraction, it was 105°. Two weeks ago, I used Hagar's dilators, with which I sufficiently dilated the os and cervix, and, with the aid of Emmet's curette forceps and Recamier's curette, cleaned out the cavity of the uterus. Hagar's dilators I can highly recommend for this purpose. You will be surprised at the ease with which you can dilate with them. Immediately after the dilatation, there was a discharge of about one ounce of a horribly offensive matter. The parts, after the scraping, were syringed out twice daily with a 1-5000 solution of corrosive sublimate. I removed from one to two teaspoonfuls of broken-down tissue. Twenty-four hours after the operation, there was decided improvement, the temperature fell, and there was but little discharge. When such symptoms occur after delivery, we are called upon to explore and remove clots, fragments of placenta, or whatever decomposing tissue may be there. But, in view of the danger I have referred to, we should be always sure that there is something to be removed before we resort to the operation. Keep the os well dilated so as not to imprison the discharge. Then, if the offensive discharge continues, you must interfere. The French doctrine is, that it is better to die of the doctor than of the disease. There is some element of truth in this idea, and when a woman is slowly and surely dying from septicæmia, the indications for the removal of the cause are very urgent, even though her chances of recovery are very slight. Dr. Parrish saw this woman and expressed the opinion that she could not live; so did I. If I had expressed such an opinion in private practice, I imagine that when the woman recovered, my services would have been no longer required in that particular family. Therefore, be very careful how you venture an opinion, for some of the most desperate cases will rally and get well. This woman was nearly pulseless, her face blue, yet she is now on the highway to recovery. Such a case should inspire us with hope and confidence. When Sydney Smith was once congratulated upon his recovery from a severe illness, and some one made the remark that he must have been very close to the gate of eternity, he answered that he was close; that he heard the hinges creak. So, in such cases, we may hear the hinges creak, but do not give up the battle; fight to the water's edge. Coleridge once said that the doctor who was most successful in treating nervous diseases was he who inspired the most hope. This is true of all diseases. Let us be ministers of hope with our patients to the very last moment; do not tell them falsehoods, for he who lies to a dying man or woman is guilty of a great crime. Cheer them up by encouraging words; but never, I say again, tell a falsehood to man, woman or child.

